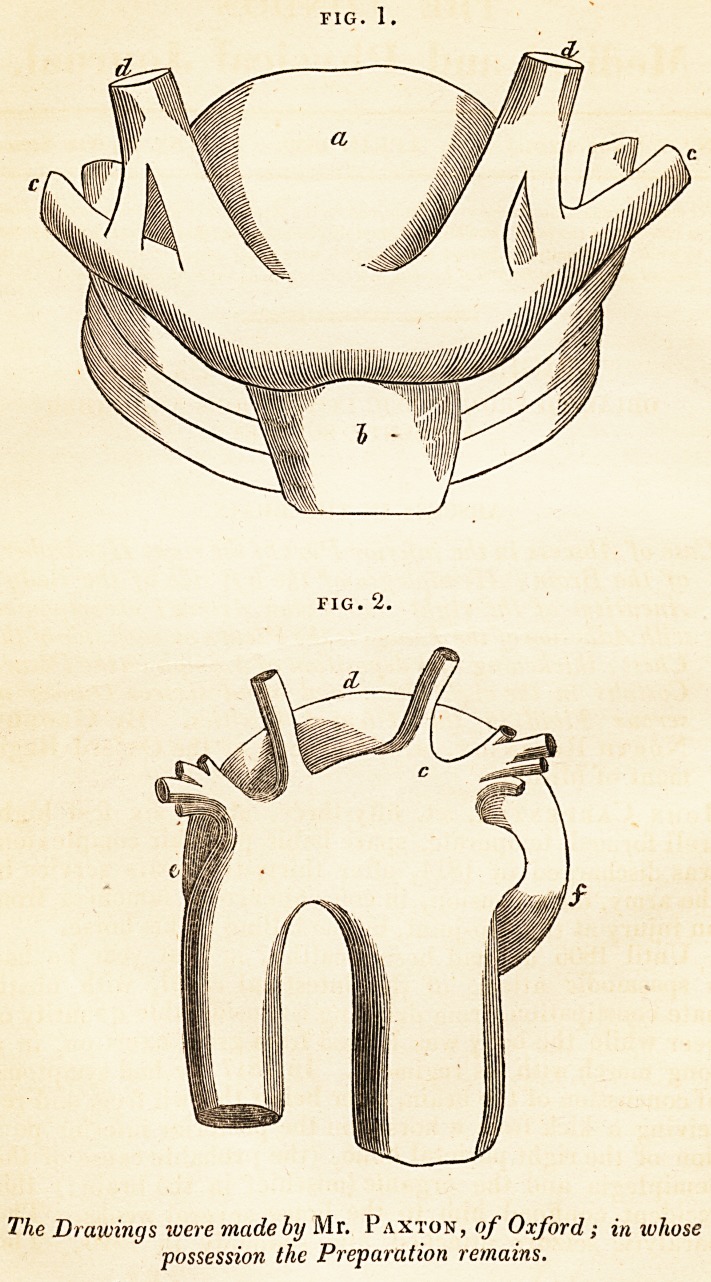# Case of Abscess in the Inferior Part of the Right Hemisphere of the Brain; Hemiplegia of the Left Side of the Body; Aneurism of the Right Subclavian Artery: Accompanied with Adhesion of the Lungs to the Pleura on Each Side of the Chest; Thickening and Deposition of Lymph on the Pleura Costalis in the Right Side; and about Sixteen Ounces of Serous Fluid in the Pleural Cavities

**Published:** 1830-04

**Authors:** George North Robinson

**Affiliations:** Surgeon of the Oxford Regiment of Militia.


FIG. 1.

FIG. 2.

*The Drawings were made by* Mr. PAXTON, *of Oxford; in whose possession the Preparation remains.*

285

THE LONDON
### Medical and Physical Journal.

No. 374, VOL. LXIII.] APRIL 1830. [No. 46, *New Series.*

For many fortunate discoveries in medicine, and for the detection of numerous errors, the world is indebted to the rapid circulation of Monthly Journals; and there never existed any work to which the Faculty, in Europe and America, were under deeper obligations than to the *Medical and Physical Journal of London*, now forming a long but an invaluable series.—*Rush.*

### ORIGINAL PAPERS, AND CASES,

### OBTAINED FROM PUBLIC INSTITUTIONS AND OTHER AUTHENTIC SOURCES.

### ABSCESS IN THE BRAIN.

*Case of Abscess in the inferior Part of the right Hemisphere of the Brain; Hemiplegia of the left side of the Body; Aneurism of the right Subclavian Artery: accompanied with Adhesion of the Lungs to the Pleura on each side of the Chest; thickening and deposition of Lymph on the Pleura Costalis in the right side; and about sixteen Ounces of serous Fluid in the Pleural Cavities.* By GEORGE NORTH ROBINSON, M.D. Surgeon of the Oxford Regiment of Militia.

JOHN CARPENTER, æt. fifty-three, about six feet high, well formed, temperate, spare habit, pale fair complexion, was discharged in 1814, after thirty-two years' service in the army, on a pension, in consequence of lameness from an injury at the hip-joint, by the falling of his horse.

Until 1806 he had been healthy: in that year he had a spasmodic attack of the intestinal canal, with obstinate constipation, from drinking a considerable quantity of beer while the body was heated from great exertion, in a long march with his regiment. In 1817 he had symptoms of concussion of the brain, after being thrown from and receiving a kick from a horse, on the posterior inferior portion of the right parietal bone, (the probable cause of the hemiplegia and the organic mischief in the brain:) this accident confined him to the house several weeks. The paralytic seizure attacked him suddenly in 1826. The aneurismal tumor was not noticed till 1827: it gradually increased, in a bilobulated, semicircular form, to the size of a moderate orange, under the sternal portion of the right clavicle, forcing it from its articulation with the sternum, and extending upwards towards the trachea. The aneurismal sac was found in a collapsed state. Had troublesome dry cough and laborious respiration latterly, accompanied with the *bruit de soufflet*, and oppressed pulse. The patient had been subject to symptoms resembling chronic catarrh about twelve years, which were doubtless owing to the gradual enlargement of the aneurismal tumor. It is a well-ascertained fact that the symptoms of thoracic aneurism, before any external swelling can be detected, often resemble those of phthisis; and the latter is sometimes actually supposed to be the disease under which the patient is labouring.

*Post-mortem appearances.* The body was emaciated, and the left arm and leg were more particularly wasted. The anterior and lower part of the neck presented a two-lobed tumor, (fig. 1, *a*,) of the size of a large orange, projecting between and above *c, c,* the clavicles. On dissecting down to these parts, and removing them for more accurate inspection, an aneurism was found to have taken place at *c*, fig. 2, the arteria innominata, which was obliterated by it, or rather had extended it to *d, e, f,* the situation just mentioned, and had caused it to assume the form here represented. The ascending aorta was enlarged, and patches of ossification were numerous in it. The inner extremities of the clavicles, and the superior part of the sternum were absorbed. As is usual in these tumors, the internal part, which was out of the line of the circulation, was filled with laminæ of coagula. The walls of the sac consist of a very firm, condensed, cellular coat, of sufficient strength to retain the clavicles in their natural line, and even to confine their disarticulated extremities in their relative situations, giving also a firm attachment to the sternal portion of *d, d,* fig. 1, the sterno-cleido-mastoideus muscles.

The brain was found to contain an excavation in its right anterior lobe, the whole of the corpora striata having disappeared, and merely a little fibrous lymph occupied its situation.

## Figures and Tables

**Fig. 1. Fig. 2. f1:**